# Rapamycin sensitizes T-ALL cells to dexamethasone-induced apoptosis

**DOI:** 10.1186/1756-9966-29-150

**Published:** 2010-11-18

**Authors:** Ling Gu, Chenyan Zhou, Huajun Liu, Ju Gao, Qiang Li, Dezhi Mu, Zhigui Ma

**Affiliations:** 1 Department of Pediatrics, West China Second University Hospital, Sichuan University, Chengdu, China

## Abstract

**Background:**

Glucocorticoid (GC) resistance is frequently seen in acute lymphoblastic leukemia of T-cell lineage (T-ALL). In this study we investigate the potential and mechanism of using rapamycin to restore the sensitivity of GC-resistant T-ALL cells to dexamethasone (Dex) treatment.

**Methods:**

Cell proliferation was detected by 3-(4,5-dimethylthiazol-2-yl)- 2,5-diphenyltetrazolium bromide (MTT) assay. Fluorescence-activated cell sorting (FACS) analysis was used to analyze apoptosis and cell cycles. Western blot analysis was performed to test the expression of the downstream effector proteins of mammalian target of rapamycin (mTOR), the cell cycle regulatory proteins, and apoptosis associated proteins.

**Results:**

10 nM rapamycin markedly increased GC sensitivity in GC-resistant T-ALL cells and this effect was mediated, at least in part, by inhibition of mTOR signaling pathway. Cell cycle arrest was associated with modulation of G_1_-S phase regulators. Both rapamycin and Dex can induce up-regulation of cyclin-dependent kinase (CDK) inhibitors of p21 and p27 and co-treatment of rapamycin with Dex resulted in a synergistic induction of their expressions. Rapamycin did not obviously affect the expression of cyclin A, whereas Dex induced cyclin A expression. Rapamycin prevented Dex-induced expression of cyclin A. Rapamycin had a stronger inhibition of cyclin D1 expression than Dex. Rapamycin enhanced GC-induced apoptosis and this was not achieved by modulation of glucocorticoid receptor (GR) expression, but synergistically up-regulation of pro-apoptotic proteins like caspase-3, Bax, and Bim, and down-regulation of anti-apoptotic protein of Mcl-1.

**Conclusion:**

Our data suggests that rapamycin can effectively reverse GC resistance in T-ALL and this effect is achieved by inducing cell cycles arrested at G_0_/G_1 _phase and activating the intrinsic apoptotic program. Therefore, combination of mTOR inhibitor rapamycin with GC containing protocol might be an attracting new therapeutic approach for GC resistant T-ALL patients.

## Background

Glucocorticoids (GCs) like prednisolone and dexamethasone (Dex) specifically induce apoptosis in malignant lymphoblasts, and therefore constitute a central role in the treatment of lymphoid malignancies, particularly acute lymphoblastic leukemia (ALL) for decades [1]. Reduction of leukemic blasts after GC administration alone has been observed in 75%-90% of newly diagnosed ALL in children and initial response to GC therapies has a strong prognostic value in ALL [2]. High sensitivity of leukemic blasts to GC determined by in vitro 3-(4,5-dimethylthiazol-2-yl)- 2,5-diphenyltetrazolium bromide (MTT) assay was also associated with good prognosis [3]. However, clinically GC resistance occurs in 10-30% of untreated ALL patients and is more frequently seen in T-lineage ALL (T-ALL) than B-precursor ALL and GC resistance always leads to the failure of chemotherapy [4]. T-ALL is a highly malignant tumor representing 10%-15% of pediatric and 25% of adult ALL in humans and it is clinically regarded as a high-risk disease with a relapse rate of about 30% [5,6]. T-ALL has a less favorable prognosis than B-cell ALL.

The mechanisms that underlie the development of GC resistance are poorly understood and likely vary with disease type, treatment regimen, and the genetic background of the patient [7]. However, an increasing number of reports indicate that activation of mammalian target of rapamycin (mTOR) signaling pathway may contribute to GC resistance in hematological malignancies [8-11]. A recent study, using a database of drug-associated gene expression profiles to screen for molecules whose profile overlapped with a gene expression signature of GC sensitivity/resistance in ALL cells, demonstrated that the mTOR inhibitor rapamycin profile matched the signature of GC sensitivity [12]. We recently demonstrated that nucleophosmin-anaplastic lymphoma kinase (*NPM-ALK*), an oncogene originated from t(2;5)(p23;q35) in a subset of non-Hodgkin's lymphoma transformed lymphoid cells to become resistant to GC or Dex treatment by activating mTOR signaling pathway and rapamycin could re-sensitize the transformed lymphocytes to Dex treatment [13].

Rapamycin, the best studied mTOR inhibitor, was originally isolated from the soil bacterium Streptomyces hygroscopicus in the mid-1970 s [14]. Although it was initially developed as a fungicide and immunosuppressant, antitumor activity of rapamycin has been described *in vitro *and *in vivo *[15-18]. mTOR is a serine-threonine protein kinase that belongs to the phosphoinositide 3-kinase (PI3K)-related kinase family. Inhibition of mTOR kinase leads to dephosphorylation of its two major downstream signaling components, p70 S6 kinase (p70S6K) and eukaryotic initiation factor 4E (eIF4E) binding protein 1 (4E-BP1), which in turn inhibits the translation of specific mRNAs involved in cell cycle and proliferation and leads to G_1 _growth arrest [19,20]. A major regulator of the mTOR pathway is the PI3K/AKT kinase cascade and activation of PI3K/AKT/mTOR has been found in lymphoid malignancies [21].

Most studies have shown that rapamycin acts as a cytostatic agent by arresting cells in the G_1 _phase [15-20]. Although cell cycle arrest can temporarily halt tumor progression, the affected clones could re-grow since the tumor cells have not been killed. Cell cycle inhibitor seems to work best in combination with chemotherapy. However, combination of cell cycle inhibitor with cytotoxic agents might be agonistic or antagonistic [22,23]. In this paper, we demonstrate that rapamycin can re-sensitize GC-resistant T-ALL cells to Dex-induced apoptosis and explore the potential therapeutic use of the selective mTOR inhibitor rapamycin for GC-resistant T-ALLs.

## Materials and methods

### Cell lines

The T-ALL cell lines, Molt-4 (GC resistant) and Jurkat (GC resistant) were kindly provided by Dr. Stephan W. Morris (St. Jude Children's Research Hospital). CEM-C1-15 (GC resistant) and CEM-C7-14 (GC sensitive) were kindly provided by Dr. E. Brad Thompson (University of Texas Medical Branch). All cell lines were maintained in RPMI 1640 (Gibco, Carlsbad, CA, USA) supplemented with 10% fetal bovine serum (FBS, Sigma, St Louis, MO, USA), 2 mM L-glutamine (Gibco), and antibiotics (penicillin 100 U/mL and streptomycin 50 μg/mL) at 37°C in a humidified 5% CO_2 _in-air atmosphere.

### Reagents and antibodies

Rapamycin (Calbiochem, La Jolla, CA, USA) was dissolved in dimethyl sulfoxide (DMSO, Sigma) and used at the concentration of 10 nM. Dex (Sigma) was dissolved in ethanol and used at the concentration of 1 μM. The final concentrations of DMSO and ethanol in the medium were 0.05% and 0.1%, respectively, at which cell proliferation/growth or viability was not obviously altered. MTT and Propidium iodide (PI) were purchased from Sigma. Annexin V-PI Kit was purchased from Keygen (Nanjing, China). Antibodies to phospho-4E-BP1, phospho-p70S6K, cyclin D1, p27, Bax, and Bcl-2 were purchased from Cell Signaling Technology (Beverly, MA, USA). Antibody to p21 was purchased from BD Bioscience (San Jose, CA, USA) and antibodies to Bim, Mcl-1, cyclin A, caspase-3 (cleaved at Asp175), NF-κB, and secondary antibodies of horseradish peroxidase (HRP)-conjugated donkey anti-rabbit antibody and HRP-conjugated sheep anti-mouse antibody were all obtained from Santa Cruz Biotech (Santa Cruz, CA, USA). Anti-GAPDH antibody was obtained from Kangchen Bio-Tech (Shanghai, China).

### Cell treatment

Logarithmically growing cells were harvested and replaced in 96- or 6-well sterile plastic culture plates (Corning Inc., Acton, MA, USA), to which 10 nM rapamycin (Rap group), 1 μM Dex (Dex group), 10 nM rapamycin plus 1 μM Dex (Rap+Dex group), and 0.05% DMSO plus 0.1% ethanol (Control group) were added respectively. At the end of the incubation period, cells were transferred to sterile centrifuge tubes, pelleted by centrifugation at 400 g at room temperature for 5 min, and prepared for analysis as described below.

### Proliferation assay

MTT assay is based on the conversion of the yellow tetrazolium salt to purple formazan crystals by metabolically active cells and provides a quantitative estimate of viable cells. Cells were seeded in 96-well plates (20,000/mL) and incubated for 48 h. 0.5 mg/mL MTT (final concentration) was added to each well for 4 h at 37°C. Then, 100% (v/v) of a solubilization solution (10% SDS in 0.01 M HCl) was added to each well, and the plates were re-incubated for 24 h at 37°C. Spectrophotometric absorbance was measured at 570 nm (reference 690 nm) using a multi-plate reader (Multiskan Spectrum, Thermo Electron Co., Vantaa, Finland). Values were obtained by comparing these cells with their respective controls.

### Cell cycle analysis

For each analysis, 10^6 ^cells were harvested 48 h after treatment and fixed overnight in 70% ethanol at 4°C. Cells were then washed and stained with 5 μg/ml PI in the presence of DNAse free RNAse (Sigma). After 30 min at room temperature, the cells were analyzed via flow cytometry (Beckman Coulter, Inc., Miami, FL, USA).

### Assay for apoptosis

The samples were washed with phosphate-buffered saline (PBS) twice and re-suspended in 500 μl of binding buffer containing 5 μl of Annexin V-FITC stock solution and 5 μl of PI for determination of phosphatidylserine exposure on the outer plasma membrane. After incubation for 10 min at room temperature in a light-protected area, the samples were quantified by flow cytometry (FASCAria, BD Bioscience, San Jose, CA).

### Western blot analysis

Cells (10^6^) were washed twice in cold PBS, and then lysed by Laemmli sample buffer (Bio-Rad, Hercules, CA, USA). Samples were boiled for 5 min at 100°C. Proteins were separated on 10% or 15% SDS-polyacrylamide gel electrophoresis (SDS-PAGE) and transferred onto nitrocellulose membranes (0.45 μm, Mllipore, São Paulo, SP, Brazil). Nonspecific-binding sites were blocked with 5% non-fat dry milk dissolved in TBS (10 mM Tris-HCl, pH 7.6, 137 mM NaCl) with 0.1% Tween 20 (TTBS) for 1 h at room temperature followed by incubation with primary antibody at 4°C overnight. The membranes were then washed 3 times in TTBS and incubated for 1 h at room temperature with secondary horseradish peroxidase (HRP)-conjugated donkey anti-rabbit antibody or HRP-conjugated sheep anti-mouse antibody diluted 1:5000 in TTBS with 5% non-fat milk. Proteins were visualized by ECL plus (Amersham Biosciences, Inc., Piscataway, NJ). All experiments were carried out independently at least 3 times. The level of the GAPDH protein was used as a control of the amount of protein loaded into each lane.

### Statistical analysis

All assays were performed in triplicate, and data are expressed as mean values ±SD. The Student's t-test was used to compare two groups. Results were considered significant with *p*-value < 0.05.

## Results

### Rapamycin and Dex inhibit growth of T-ALL cells synergistically

**Figure 1:**
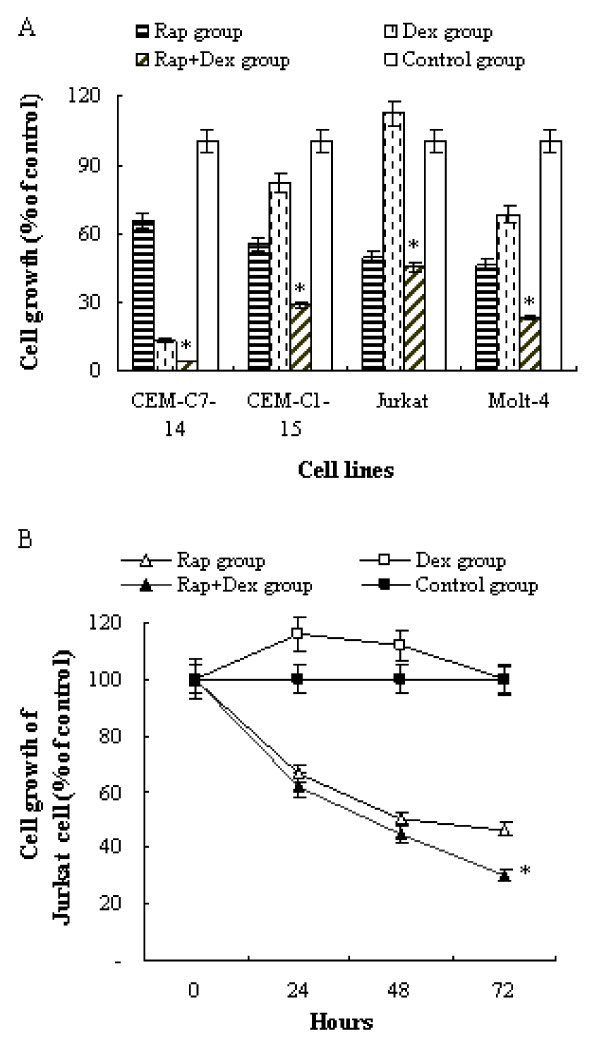
**Rapamycin augments Dex's growth inhibition on T-ALL cell lines**. (A) Four T-ALL cell lines (CEM-C7-14, CEM-C1-15, Molt-4, and Jurkat) were incubated for 48 h with rapamycin (10 nM) and/or Dex (1 μM), and the proliferation rate of the cells were evaluated by MTT assay. (B) GC-resistant cell line Jurkat was exposed for 72 h to rapamycin (10 nM) and Dex (1 μM) alone or in combination. At time 0, 24, 48 and 72 h after treatment, proliferation rate of the cells were evaluated by MTT assay. For each assay, values of triple experiments were shown as mean plus or minus SD. * *p *< 0.05 as compared with control group or Rap group or Dex group.

It has been reported that rapamycin can sensitize multiple myeloma cells to apoptosis induced by Dex [9,11]. In order to evaluate the potential of rapamycin for the treatment of GC-resistant ALL, we selected a panel of four T-ALL cell lines, GC-sensitive CEM-C7-14, and the GC-resistant CEM-C1-15, Molt-4, and Jurkat. Four cell lines were incubated for 48 h with rapamycin and/or Dex. Rapamycin inhibited the growth of all the four T-ALL cell lines. The percentage of viable cells were from the lowest of 46% in Molt-4 to the highest of 66% in CEM-C7-14 as compared to their control group, *p *<*0.05*. The response of the T-ALL cell lines to Dex varied. The GC-sensitive cell line CEM-C7-14 was highly sensitive to GC with only 13% of the cells viable. The other cell lines were GC resistant, with the viability from the lowest of 69% in Molt-4 to the highest of 112% in Jurkat. However, combination of rapamycin with Dex strongly enhanced the growth inhibitory effect on Molt-4, CEM-C1-15, and CEM-C7-14 cells compared with single use of rapamycin or Dex, *p *< 0.05 (Figure 1A). Although co-treatment of rapamycin with Dex did not show a stronger growth inhibition compared with singly use of rapamycin at 48 h in Jurkat cells, there was an obvious difference on the growth inhibition after 72 h. The cell viability was 45% in the former versus 31% in the later, *p *< 0.05 (Figure 1B). These data suggested that rapamycin and Dex had synergistic growth inhibition on T-ALL cells.

### Rapamycin and Dex acts synergistically on the inhibition of mTOR signaling pathway

**Figure 2:**
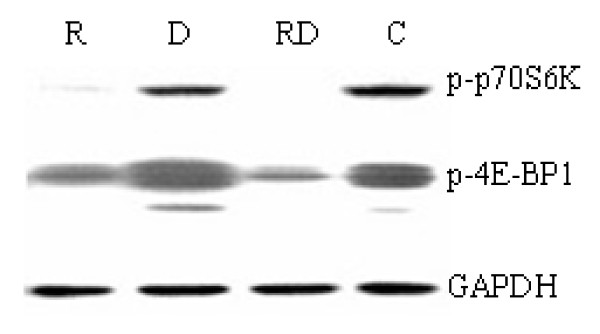
**The effect of rapamycin and Dex on mTOR pathway**. Molt-4 cells were treated with rapamycin and/or Dex. After 48 h, cells were lysed and followed by Western blot analysis using antibodies specific for the p70S6K phosphorylation sites Thr421/Ser424 and 4E-BP1 phosphorylation sites Thr37/46.

Rapamycin inhibits cell grow through dephosphorylation of p70S6K and 4E-BP1 [15-20]. The phosphorylation status of p70S6K and 4E-BP1 is commonly employed to assess the inhibition of mTOR by rapamycin. We performed Western blot analysis using antibodies specific for the p70S6K phosphorylation sites Thr421/Ser424 and 4E-BP1 phosphorylation sites Thr37/46 in Molt-4 cells. Just as expected, rapamycin inhibited phosphorylation of both p70S6K and 4E-BP1 (p-p70S6K and p-4E-BP1). Dex alone had no effect on p-p70S6K and p-4E-BP1. However, when combined use of these two drugs, a synergistic inhibition of mTOR signaling was detected by de-phosphorylation of p70S6K and 4E-BP1 (Figure 2). These results suggested that inhibition of the mTOR signaling pathway may potentiate the cytotoxic effect of Dex. The same results were obtained in both Jurkat and CEM-C1-15 cells (data not shown).

### Rapamycin and Dex arrest T-ALL cells in G_0_/G_1 _phase of the cell cycle

**Figure 3:**
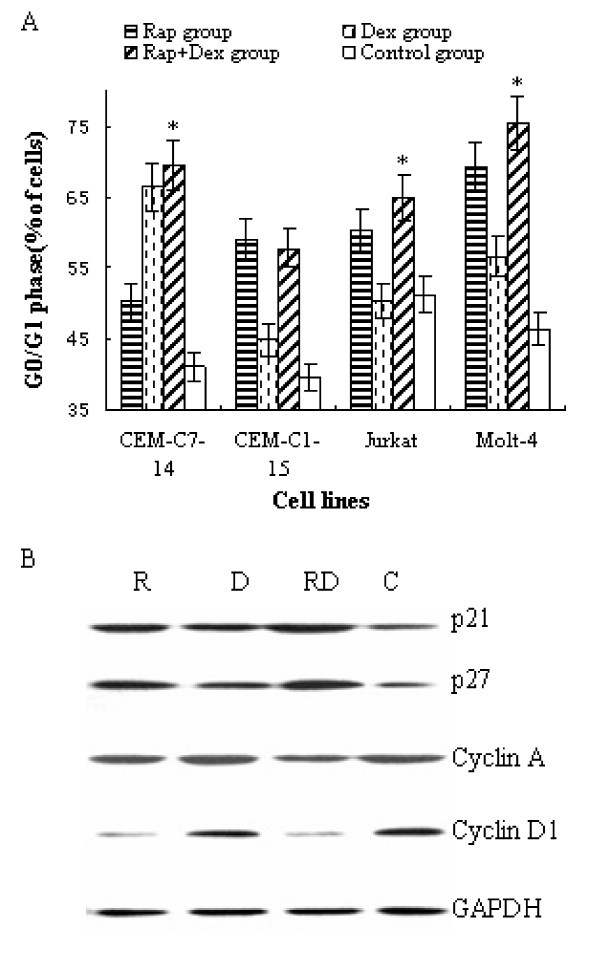
**The effect of rapamycin and Dex on cell cycles and the cell cycle regulatory proteins**. (A) T-ALL cells were incubated for 48 h with rapamycin(10 nM) and/or Dex (1 μM) and the cell cycle phases were analyzed by PI staining. For all experiments, values of triple experiments were shown as mean plus or minus SD. * *p *< 0.05 as compared with control group or Dex group or Rap group except for CEM-C1-15 cells. (B) Cell-cycle proteins were studied. After 48 h exposure to rapamycin and/or Dex, Molt-4 cells were lysed and extracts were analyzed by Western blotting. R, rapamycin; D, Dex; RD, rapamycin+Dex; and C, control.

The main role of rapamycin is to induce cell cycle arrest [19,20]. Flow cytometric analysis showed that 48 h treatment with rapamycin clearly induced G_0_/G_1 _arrest in all 4 cell lines of T-ALL. In GC-sensitive cell line, CEM-C7-14, Dex itself, can induce G_0_/G_1 _arrest, and co-treatment with rapamycin increased the G_0_/G_1 _phase slightly, from 67% to 70%, p > 0.05. But in GC-resistant cell lines, rapamycin augmented the effect of G_0_/G_1 _arrest significantly, from 45% to 58% in CEM-C1-15 cells, 50% to 65% in Jurkat cells, and 57% to 75% in Molt-4 cells, *p *<*0.05 *(Figure 3A).

To evaluate the molecular basis underlying cell cycle arrest, we investigated the expression of cell cycle regulatory proteins. As shown in Figure 3B, both rapamycin and Dex could induce up-regulation of cyclin-dependent kinase (CDK) inhibitors of p21 and p27, and a synergistic effect of induction was detected when using these two drugs together. Rapamycin did not obviously affect the expression of cyclin A, whereas dexamethasone induced cyclin A expession. Rapamycin prevented dexamethasone-induced expression of cyclin A. Cyclin D1 levels were reduced when treated with rapamycin or dexamethasone alone, or in combination. Compared with Dex, rapamycin had a stronger effect on down-regulation of cyclin D1.

### Rapamycin sensitizes T-ALL cells to Dex-induced apoptosis

**Figure 4:**
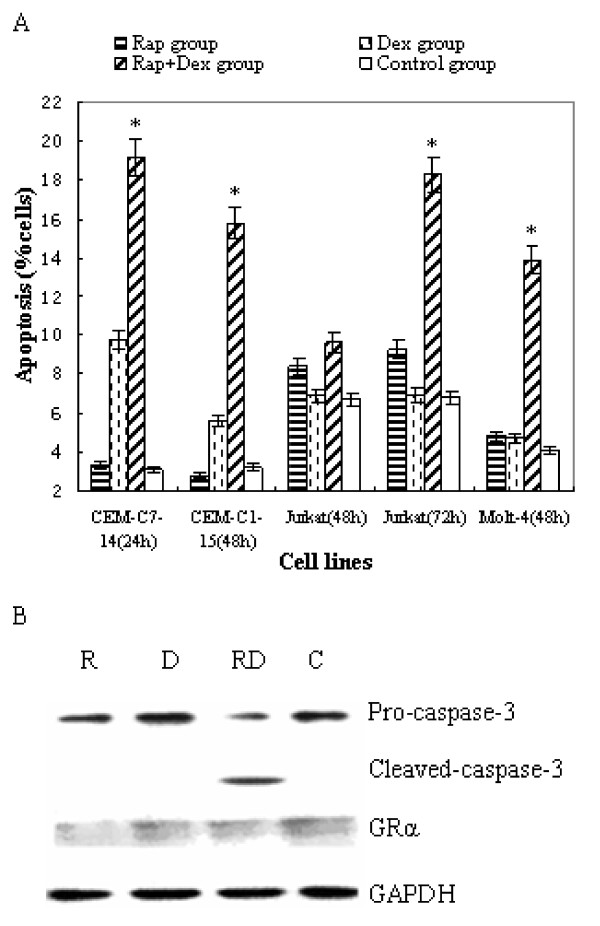
**Rapamycin sensitizes T-ALL cells to GC treatment by enhancing apoptotic cell death**. (A) T-ALL cells were incubated for 24~72 h (according to different time points to early stage of apoptosis) with rapamycin(10 nM) and/or Dex (1 μM), and the early stage of apoptosis were detected by Annexin V-FITC/PI staining. For all experiments, values of triple experiments were shown as mean plus or minus SD. * *p *< 0.05 as compared with control group or Dex group or Rap group (except for Jurkat cells at 48 h). (B) After 48 h exposure to rapamycin and/or Dex, Molt-4 cells were lysed and extracts were analyzed by Western blotting for GR expression.

Cell cycle arrest could not explain the magic effect on growth inhibition of Dex when co-treated with rapamycin. The main mechanism of Dex in the treatment of lymphoid malignancies is to induce apoptotic cell death. We used Annexin V-PI staining to determine the early stage of apoptosis. Dex, used alone at 1 μM (Dex group), had no apoptotic effect on Jurkat and Molt-4 cells, and there was only a minimal effect on CEM-C1-15 cells at 48 h and a modest effect on CEM-C7-14 cells at 24 h (After 24 h the cells came to the late phase of apoptosis, data not shown.), *p *>*0.05*. Rapamycin, used at 10 nM (Rap group), also had no obvious apoptosis-inducing effect on all 4 cell lines, although at this concentration, significant cell cycle arrest at G_1 _phase occurred (Figure 3A). However, when combined Dex with rapamycin (Rap+Dex group), a remarkable increase in cell apoptosis was ensued in all 4 cell lines (Figure 4A). Compared with Rap group, the combination treatment group of cells increased the apoptotic rate from 3% to 20% in CEM-C7-14 at 24 h, *p *<*0.05*, from 3% to 16% in CEM-C1-15 cells at 24 h, *p *<*0.05*, from 9% to 18% in Jurkat cells at 72 h, *p *<*0.05*, and from 5% to 14% in Molt-4 cells at 48 h, *p *<*0.05*. Taken together, these results suggest that rapamycin can augment the cytotoxic effect of Dex in both GC-sensitive and resistant cells.

**Figure 5:**
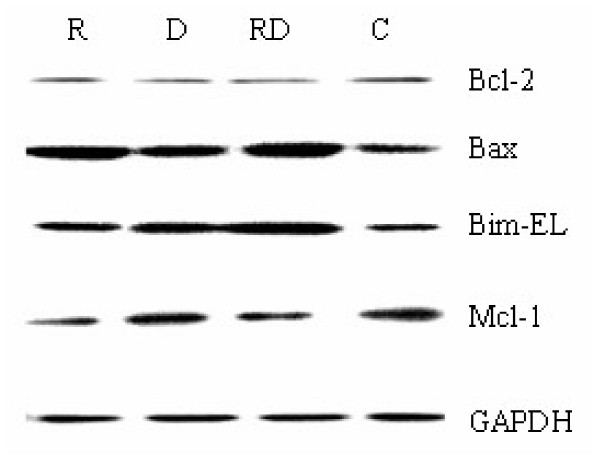
**Western blot analysis of the apoptosis associated proteins in Molt-4 cells after 48 h exposure to rapamycin and/or Dex**. R, rapamycin; D, Dex; RD, rapamycin+ Dex; and C, control.

The ability to up-regulate glucocorticoid receptor (GR) expression upon GC exposure has been demonstrated in various cell lines of lymphoid leukemias and this up-regulation of GR has been suggested as an essential step to the induction of apoptosis in leukemic cells [24]. In Molt-4 cells, we found no change of GR expression after treatment with rapamycin or Dex singly or in combination (Figure 4B). So up-regulation of GR expression might not participate in the mechanism of rapamycin's reversion of GC resistance in GC-resistant T-ALLs. In the same cells, we found that although caspase-3 was not activated by rapamycin or Dex alone, but a strong activation was ensued after combined treatment (Figure 4B), suggesting that apoptosis mechanism did involve in the process. We then examined the expressions of Bcl-2, Bax, Bim-EL, and Mcl-1 in Molt-4 cells. Similar to other study [12], levels of the anti-apoptotic protein Bcl-2 was unchanged after exposure to rapamycin or Dex alone or in combination, whereas Mcl-1 level was reduced significantly after exposure to rapamycin alone or in combination with Dex, but not modulated by Dex alone. Both Dex and rapamycin induced expression of Bim-EL and Bax significantly and there was a synergistic effect when they were used together (Figure 5). These data further support that rapamycin reverses GC resistance via activation of the intrinsic apoptotic program.

## Disccusion

In vivo response to 7 days of monotherapy with prednisone is a strong and independent prognostic factor in childhood ALL [25]. Despite intensive research efforts, GC resistance remains a major obstacle to successful T-ALL treatment. Increasing evidences now indicate that rapamycin, the mTOR inhibitor, could be used as a potential GC sensitizer [9-13]. In this study, we wanted to explore the possibility of using rapamycin as a therapeutic element in the GC-resistant T-ALLs. Our results showed that Dex had minimal effects on the cell growth and apoptosis of the GC-resistant T-ALL cell lines, but when it was used to co-treat the cells with rapamycin, a stronger growth inhibitory and apoptosis-inducing effect was achieved and it was done through synergistically inhibiting mTOR signaling, suggesting a rationale of adding mTOR inhibitor in the treatment of GC resistant T-ALLs in clinics.

Down-regulation of cyclin D1 along with up-regulation of CDK inhibitors p21 and p27 have previously been suggested to be the mechanism behind mTOR inhibitor induced cell cycle arrest [26,27]. We got the same results in GC-resistant Molt-4 cells. We also found that compared with rapamycin treatment alone, combined treatment with Dex decreased the expression level of cyclin A, which would also contribute to the effect of cell cycle arrest at G1 phase.

It's an exciting finding that rapamycin can reverse GC resistance in T-ALL cell lines, although the exact mechanism of GC resistance has poorly understood yet. GC resistance may caused by lack of GR up-regulation upon GC exposure in leukemia cell lines [28]. However, evidence showed that GC resistance in childhood ALL cannot be attributed to an inability of resistant cells to up-regulate the expression of the GR upon GC exposure, nor to differences in *GR *promoter usage [24]. Our studies demonstrated that rapamycin's reversion of GC resistance in T-ALLs was not through modulation of GR expression.

Bcl-2 family members are critical regulators of the intrinsic apoptotic pathway and play critical roles in GC-induced apoptosis [29]. The members of this family can be divided into two groups, the anti-apoptotic proteins, such as Bcl-2 and Mcl-1, and the pro-apoptotic proteins, such as Bax and Bim. The down-regulation of Mcl-1 was recently shown to be critical for sensitizing GC-induced apoptosis in lymphoid malignancy cells [12]. Our studies showed that in Molt-4 cells rapamycin can inhibit Mcl-1 and rapamycin and Dex have a synergistic induction of Bax and Bim, suggesting that rapamycin sensitizes GC-induced apoptosis in T-ALL cells by modulation of apoptosis related proteins.

In conclusion, we show in this study that rapamycin enhances Dex induced apoptosis by inhibition of mTOR signaling pathway and activation of the intrinsic apoptotic program. Clinical trials of rapamycin and its derivates have been completed or are ongoing for the treatment of hematologic malignancies [21]. Therefore, combination of these drugs with current ALL protocols might be an attracting new therapeutic approach for GC-resistant T-ALL patients.

## Competing interests

The authors declare that they have no competing interests.

## Authors' contributions

LG and CZ designed the experiments, conducted the studies, prepared all the figures, and drafted the manuscript. HL and QL participated in data analyses, interpretation of results, and checking the manuscript for typographical errors. JG and DM participated in the design of the study and carried out data interpretation. ZM contributed to conception, experimental design, data acquisition, analyses, and interpretation, and manuscript preparation. All authors read and approved the final manuscript.
